# Association between Polymorphisms of Antioxidant Gene (MnSOD, CAT, and GPx1) and Risk of Coronary Artery Disease

**DOI:** 10.1155/2018/5086869

**Published:** 2018-08-26

**Authors:** Hseng-Long Yeh, Li-Tang Kuo, Fung-Chang Sung, Chih-Ching Yeh

**Affiliations:** ^1^School of Public Health, College of Public Health, Taipei Medical University, Taipei, Taiwan; ^2^Division of Cardiology, Department of Internal Medicine, Sijhih Cathay General Hospital, Sijhih City, Taipei, Taiwan; ^3^Division of Cardiology, Department of Internal Medicine, Chang Gung Memorial Hospital, Keelung, Taiwan; ^4^Chang Gung University College of Medicine, Taoyuan, Taiwan; ^5^Management Office for Health Data, China Medical University Hospital, Taichung, Taiwan; ^6^Graduate Institute of Clinical Medical Science, School of Medicine, College of Medicine, China Medical University, Taichung, Taiwan; ^7^Department of Public Health, China Medical University, Taichung, Taiwan

## Abstract

**Objective:**

Reactive oxygen species (ROS) been cited as one of the major causes of atherosclerosis and coronary artery disease which are possible agents inducing DNA damage. Manganese superoxide dismutase (MnSOD), catalase (CAT), and glutathione peroxidase-1 (GPx1) have evolved to address primary defense against free radical mediated damage in mitochondria. The aim of this study was to delineate the association of* MnSOD*,* CAT*, and* GPx1* polymorphisms and risk of CAD in Taiwan.

**Methods:**

We conducted a case-control study with 657 participants recruited at a medical center. All subjects were evaluated by noninvasive stress test and then quantitative coronary angiography to confirm the diagnosis of CAD. 447 CAD cases were defined as >50% stenosis of coronary artery and 210 controls were stenosed below 50%. Polymorphisms of* MnSOD* (Val16Ala),* CAT* (C-262T), and* GPx1* (Pro198Leu) genes were determined by polymerase chain reaction methods. Multivariate logistic regression model was used to calculate the odds ratios (ORs) and 95% confidence intervals (CIs).

**Results:**

The* MnSOD* Val/Ala+Ala/Ala genotype was significantly associated with an increased risk of CAD compared to the Val/Val genotype (OR = 1.86, 95% CI = 1.15-3.01). This polymorphism was also associated with the severity of CAD of single and two vessel diseases. The corresponding ORs were 2.31 (95% CI = 1.32-4.03) and 1.92 (95% CI = 1.02-3.61), respectively. Among cigarette smokers, the harmful genetic effect of* MnSOD* Ala allele on CAD risk was much higher (OR = 2.23, 95% CI = 1.02-4.88). However, the interaction between* MnSOD* genotype and cigarette smoking on CAD risk was not significant. No significant association between* CAT* and* GPx1* polymorphisms and CAD risk was observed.

**Conclusion:**

Our results suggest that* MnSOD* polymorphism is an independent risk factor for susceptibility to CAD in the Chinese population.

## 1. Introduction

Cardiovascular disease (CVD) is the leading cause of mortality worldwide. Based on the World Health Organization (WHO) data in 2013, an estimated one-third of global deaths were attributable to CVDs [1, 2]. Through large-scale epidemiologic studies, several conventional risk factors for CVDs have been identified for instance, cigarette smoking, hypertension, hyperlipidemia, diabetes mellitus, and obesity [3]. An intensified intervention strategy, treating multiple risk factors to target, brings about a 20 percent reduction in cardiovascular events in high risk individuals [4].

However, some discovered that these well-known risk factors merely account for 50-80% of CVDs [5, 6] and still around 15-20% of myocardial infarction occurred in individuals lacking any of the traditional risks [5]. Further studies to investigate novel CVDs risk markers and thereby early recognizing preclinical disease are essential.

Since the initial human genome sequencing in 2001 [7] and publication of HapMap in 2005 [8], the development in genetic analysis and personalized precision medicine have rapidly progressed. Efforts on genetic basis for CVDs have been made, including genome-wide association study [9], but suffered from inconsistent results and still have a long way to go to make a conclusion on how genetic factors affect CVDs. Our study group had previously recruited a total of 458 CAD patients and 209 health subjects and found a null association between glutathione-S-transferase genes and susceptibility to CAD [10].

Since mitochondria make up a large mass, approximately 30% of the myocyte, oxidative stress to mitochondria plays important roles in pathophysiology of CVDs [11]. Oxidative stress might come from overproduction of ROS or inadequate antioxidant functions. MnSOD, CAT, and GPx1 are mitochondrial enzymes, which act on the ROS metabolism and participate in the cellular defence to oxidative stress [12]. The primary antioxidant in the mitochondria that is MnSOD and CAT converts ROS into oxygen and hydrogen peroxide. The second line of enzymatic antioxidant defense is GPx1 isoenzymes. To make further investigation on genetic predispositions of CAD, we have conducted a research on antioxidant genes polymorphism and CAD risk. The aim of the study is to investigate the association of SNPs such as* MnSOD*,* CAT*, and* GPx1* with risk of CAD among a population from Taiwan.

## 2. Materials and Methods

### 2.1. Subjects

Detailed descriptions of the specific characteristics of the study participants have been published previously [13]. In brief, participants were recruited from the Chang Gung Memorial Hospital Taiwan. The CAD participants in this study were angiographically confirmed by experienced cardiologists. Individuals with at least one coronary artery diameter stenosis ≥ 50% were defined as cases (n = 481) and the others as control group (n = 228) [14]. This study protocol had been proven by The Institutional Review Board at the Chang Gung Memorial Hospital and all patients provided informed consents.

Before receiving the angiography, each participant completed a self-administered questionnaire covering sociodemographic characteristics, tobacco and alcohol use, height, weight, family CAD history, and personal medical history of hypertension and diabetes. Individuals with blood pressure ≥ 140/90 mmHg or receiving antihypertensive therapy were considered as hypertensive. Those with fasting plasma glucose > 126 mg/dl or receiving glucose lowering treatment were considered as diabetes. Body mass index (BMI) was calculated as weight/height^2^ (kg/m^2^). Cigarette smoking and alcohol drinking were classified into three groups: current users, nonusers, and ex-users (who had stopped use for at least six months). Blood samples of overnight fast (12 to 14 hours) provided by subjects were measured for the biochemical profiles by the Clinical Chemistry Department at the hospital, including total cholesterol, high-density lipoprotein cholesterol (HDL-C), uric acid, creatinine, and triglyceride using standard enzymatic methods [15]. The low-density lipoprotein cholesterol (LDL-C) levels were calculated from the Friedewald formula. Non-HDL-C was equal to the level of cholesterol minus HDL-C. Triglyceride-rich lipoprotein cholesterol (TRL-C) was calculated by total cholesterol minus levels of LDL-C and HDL-C.

### 2.2. Genotyping

Genomic DNA was extracted from buffy coat cells using a DNA extraction kit (Qiagen, Inc., Chatsworth CA). The* MnSOD* Val-9Ala (rs4880),* CAT* C-262T (rs1001179), and* GPx1* Pro198Leu (rs1050450) polymorphisms were determined using polymerase chain reaction (PCR) and restriction fragment length polymorphism (RFLP) methods [16, 17]. All PCR reactions were performed in a 20 *μ*l final volume containing 0.25 *μ*M of each primer, 50 ng genomic DNA, 1.5 mM MgCl_2_, 0.2 mM dNTPs, and 1.0 unit of Taq DNA Polymerase in the buffer provided by the manufacturer. Amplification was performed in a Mastercycler gradient thermocycler (Eppendorf, Hamburg, Germany) for the PCR reaction.

The 112 bp* MnSOD* Val-9Ala PCR products were amplified with the primers 5′-GCA CCA GCA GGC AGC TGG CGC CGG-3′ (sense) and 5′-TGC GCG TTG ATG TGA GGT TCC AG-3′ (antisense) and digested with* NgoM*IV (New England BioLabs, Beverly, MA, USA). The Ala allele revealed 90 and 22 bp fragments following digestion and polyacrylamide gel electrophoresis, whilst the Val allele was not digested by* NgoM*IV [18]. The 183 bp* CAT* C-262T PCR products were amplified with the primers 5′-TAA GAG CTG AGA AAG CAT AGC T-3′ (sense) and 5′-AGA GCC TCG CCC CGC CGG ACC G-3′ (antisense) and digested with* SmaI* (Fermentas, Glen Burnie, MD, USA). The C allele revealed 153 and 30 bp fragments following digestion and polyacrylamide gel electrophoresis, while the T allele was not digested by* Sma*I [19]. The 337 bp* GPx1* Pro198Leu PCR products were amplified with the primers 5′-TGT GCC CCT ACG GTA CA-3′ (sense) and 5′-CCA AAT GAC AAT GAC ACA GG -3′ (antisense) and digested with* Apa*I (Fermentas, USA). The Pro allele revealed 258 and 79 bp fragments following digestion and polyacrylamide gel electrophoresis, whilst the Lys allele was not able to be digested by* Apa*I. For quality control, 10% replicate samples were included with the study samples and showed 100% concordance for all polymorphisms. As well, all laboratory personnel were blinded to the CAD status of the participants.

### 2.3. Statistical Analysis

A total of 52 subjects were excluded because of genotyping failure (34 cases, 18 controls), leaving 447 cases and 210 controls for the final analysis. Tests for Hardy-Weinberg equilibrium amongst controls were conducted using Chi-square test featuring one degree of freedom. Differences between cases and controls were compared by either Student's* t*-test or Wilcoxon rank-sum test for continuous variables or Chi-square test for qualitative data. Unconditional logistic regression was used to calculate the odd ratios (ORs) and 95% confidence intervals (CIs) for the CAD risk associated with the* MnSOD, CAT*, and* GPx1* genotypes. The adjusted OR of genotypes was calculated using a multivariate logistic regression model adjusted for age, sex, cigarette smoking (never and ever), and alcohol drinking (never and ever). Interaction analysis based on a multiplicative scale was conducted to evaluate the gene-environment interaction for the CAD risk. All analyses were performed using the Statistical Analysis System version 9.1.3 (SAS Institute, Cary, NC) and all tests were 2-sided with P-value < 0.05 as the significant level.

## 3. Results

The demographic and clinical characteristics of the participants in the study are summarized in Table 1. Compared with the controls, the CAD cases were primarily male, older, smokers, and presenting hypertension and diabetes (p < 0.05). The study cases also had higher levels of creatinine, uric acid, LDL-C, cholesterol, non-HDL-C, TRL-C, and triglyceride, but lowered HDL-C levels.

The genotypic distributions of the* MnSOD, CAT*, and* GPx1* polymorphisms for both cases and controls are shown in Table 2. The allele frequencies for the* MnSOD* Ala allele,* CAT* T allele, and* GPx1 *Leu allele amongst the controls were 7.38, 3.1, and 4.52%, respectively. All these polymorphisms are in Hardy-Weinberg equilibrium. Because of the low frequencies of variant alleles for these three polymorphisms, we divided each polymorphism into wild and variant genotype for further data analyses (i.e., Val/Ala+Ala/Ala and Val/Val for* MnSOD*, C/T+T/T and T/T for* CAT*, and Pro/Leu and Pro/Pro for* GPx1*). After controlling for covariates, the CAD risk was statistically significantly increased by* MnSOD* Val/Ala+Ala/Ala genotype (OR = 1.86, 95%CI = 1.15-3.01), compared to* MnSOD* Val/Val genotype. A moderately increased risk for CAD was also observed in subjects with* CAT* C/T+/T/T genotype than those with C/C genotype (OR = 1.51, 95%CI = 0.77-2.95). However, the* GPx1* polymorphism was not associated with CAD risk.

We also categorized all study subjects into four groups according to their CAD severity. As shown in Table 3, CAD risk is most profoundly increased with* MnSOD* Val/Ala+Ala/Ala polymorphisms in the one-vessel disease group (OR = 2.31, 95% CI = 1.32-4.03). Subjects with* MnSOD* Val/Ala+Ala/Ala polymorphisms also showed higher CAD risk, although less significant, in those with two-vessel disease (OR = 1.92, 95% CI = 1.02-3.06). There is no difference in CAD risk between wild and variant* MnSOD* genotypes among the three-vessel disease group (OR = 1.40, 95% CI = 0.75-2.61).

The interactions between* MnSOD, CAT*, and* GPx1* polymorphisms and cigarette smoking for CAD risk are shown in Table 4. The increased CAD risk by* MnSOD* Val/Ala+Ala/Ala genotype was pronounced among ever smokers, but this interaction was not significant (p for interaction = 0.552). Among the ever smokers, subjects carrying the* MnSOD* Val/Ala+Ala/Ala genotype had 2.23-fold risk for CAD than those carrying the Val/Val genotype (OR = 2.23, 95% CI = 1.02-4.88). However, there was no significant interaction between* CAT* and* GPx1* polymorphisms and cigarette smoking

The smoking-stratified analysis for association between* MnSOD* genotypes and CAD severity was shown in Table 5. We found that variant* MnSOD* genotypes are associated with significantly increased CAD risk only in ever cigarette smokers with one vessel disease (OR = 2.63, 95% CI = 1.07-6.44). CAD risk differences are neither seen in never cigarette smoking individuals, nor in ever smokers with more severe CAD.

## 4. Discussion

Atherosclerosis is focal thickening plaques located in coronary artery intima. A growing body of evidence shows that oxidative stress directly induce inflammation cascade and accelerate oxidation of LDL-C, causing atherosclerotic plaque instability and finally worsening of CAD [20]. Oxidative stress results from imbalance between ROS production and antioxidants capacity. Antioxidants defend cells against damage from free radicals and ROS, maintaining redox homeostasis. MnSOD, GPx1, and CAT are all endogenous enzymatic antioxidants [21, 22]. In the current study, we focus on whether genetic polymorphisms of* MnSOD*,* GPx1*, and* CAT* affect vulnerability or severity of CAD in Taiwan. Our study demonstrated a significantly elevated CAD risk in subjects with* MnSOD* Val/Ala+Ala/Ala genotype, compared to those carrying Val/Val genotype. Noticeably, among the ever-smoker subgroup, CAD risk in* MnSOD* Val/Ala+Ala/Ala genotype patients increased even further.

MnSOD is the major mitochondrial antioxidant enzyme [21]. Earlier animal studies found MnSOD knock-out Drosophila failed to survive [23].* MnSOD* Val-9Ala is a well-known polymorphism. Substitution for Alanine with Valine causes a conformational change in the mitochondrial-targeting domain and damages the enzymes function [21, 24]. This polymorphism has been linked to several diseases, including breast cancer, lung cancer, prostate cancer, dyslipidemia, macular diseases, neuropathies, and Parkinson's disease [25–27]. This implicates the importance of oxidative injury in the underlying pathophysiology of these disease processes.

The correlation between atherosclerosis and* MnSOD* polymorphism was also observed. Kakko et al. collected a sample of 989 middle-aged hypertensive and control Caucasians subjects in Finland. They found that* MnSOD* Val/Val carriers were associated with higher carotid intimal media thickness, which indicates more severe atherosclerosis [28]. Souiden et al. discovered an approximately twofold risk of CAD in Tunisian men harbouring the Val/Val genotype [22]. These results are inconsistent with our data. Among these studies, the Val allele brings about increased atherosclerotic risk, while our data demonstrated a nearly twofold risk for CAD in those harbouring Ala alleles. However, another pulmonary artery hypertension study in Chinese population found results similar to ours [29]. This inconsistency may be due to the influence of interracial* MnSOD* Ala allele frequency difference. The higher frequency of Ala allele was found as 48.3% in Caucasians and 25.1% in Arabs, respectively [22, 28]. It was a discrepancy compared to our Chinese population which was 7.83%. Therefore, the association between* MnSOD* genetic variants and CAD risk may be influenced by ethnicity.

As mentioned earlier, numerous clinical or animal studies disclosed correlations between* MnSOD* polymorphism and diseases. However, when looking into details, the results were actually inconsistent and confusing. Whether the genetic variant brings about extra risk or benefit for disease varies across races, ethnics, and disease entities [30]. For instance, the Val allele has been shown to increase cardiovascular risks in most studies, but is probably protective to Parkinson's disease. Although seemingly incoherent with other study results, our study provides a scope of how* MnSOD* Val-9Ala affects CAD in the local Taiwanese population and demonstrates a probable interaction with cigarette smoking.

GPx1 is another intracellular antioxidant. Its deficiency is linked to atherogenesis [31]. GPx1 activity is also independently associated with occurrence of CAD [32, 33].* GPx1 *Pro198Leu is the best characterized polymorphism. Souiden et al. studied 164 patients with established CAD and 203 healthy controls and revealed a null association between* GPx1* polymorphism and CAD incidence and severity [22]. The discovery from Souiden et al. is in concert with our data. In contrast, Nemoto et al. reported that type 2 diabetes mellitus subjects carrying* GPx1 *Pro/Leu genotype have significantly higher calcium score than those with Pro/Pro genotype [34]. In Nemoto's study, the subjects were mainly type 2 diabetes patients. In comparison, only 11% in control group and 38% in CAD group have been diagnosed with DM in our population. Further studies are needed to clarify if there is association between DM and* GPx1 *polymorphism.

CAT is another major defense against oxidative stress [35]. Similarly,* CAT* polymorphism has been found to be correlated with several disease entities, including cancer, and metabolic diseases, etc. [36–38]. A meta-analysis analysed 10 case-control studies and concluded that CAT activity was associated inversely with CAD [39]. However, previous genetic analysis showed no differences in allele frequencies among diabetic patients with and without cardiovascular disease [40, 41]. This is similar to our findings. However, the result may have been affected by difference of diabetes proportion since diabetes itself is an immense bias in genetically testing for CAD risk. The null association requires further confirmation.

The strength of our study is that all participants received coronary angiography to document CAD status. However, the subjects were enrolled from a single institute in Taiwan and probably represent only the local population. Besides, through angiography, we could only record the coronary vessel anatomy, but not physiological as fractional flow reserve. Therefore, latent CAD might be erroneously classified as healthy control. Current evidence about* MnSOD*,* GPx1*, and* CAT* polymorphisms are mostly observational, cross-sectional studies. To achieve better understanding on how antioxidants gene polymorphisms affect CAD, further well-conducted, large-scale, randomized control study is mandatory. In the meanwhile, the development of CAD is complex, requiring not only genetic predisposition, but also environmental factors. As shown in our study, cigarette smoking might interact with* MnSOD* polymorphism, causing a higher risk in the ever-smoking subgroup. Future study may focus more on the interaction between* MnSOD, GPx1*, or* CAT* polymorphism and other environmental risk factors. The data of apoB-100 was not included which play an important role in the process of atherosclerosis. The interaction of apoB-100 and genetic polymorphisms is not investigated in our study. Data from different races and ethnic groups should also be analysed independently.

## 5. Conclusions

Our results suggest* MnSOD* Val/Ala+Ala/Ala genotypes are independent risk factors for susceptibility to CAD in the Taiwan population. There are no significant correlations between* CAT* (C-262T) and* GPx1* (Pro198Leu) polymorphisms and CAD risk. Future large-scale, controlled trials are needed to further understand the interactions between these antioxidant gene polymorphisms and environmental factors. Local data in different ethnic groups are also important.

## Figures and Tables

**Table 1:** Demographic and clinical characteristics of the study population.

Variables^a^	Cases, n (%) N=447	Controls, n (%) N=210	p-value^b^
Male	329(73.6)	130(61.9)	0.002
Age (mean ± SD), years	64.8±12.2	58.1±12.6	<0.001
BMI (mean ± SD), kg/m^2^	25.3±4.03	25.5±3.8	0.546
Stenosis 1 / 2 / 3	159/129/159		
Ethnic group			0.591
Taiwanese	336(76.9)	165(79.7)	
Mainland Chinese	59(13.5)	27(13.0)	
Other^c^	42(9.6)	15(7.3)	
Occupation			0.390
White-collar	73(17.1)	27(13.0)	
Blue-collar	141(32.9)	69(33.2)	
Other^d^	214(50.0)	112(53.8)	
Family CAD history	104(24.9)	44(23.0)	0.508
History of hypertension	229(51.2)	84(40.0)	0.007
History of DM	173(38.7)	24(11.4)	<0.001
Cigarette smoking			<0.001
None	194(43.5)	129(61.4)	
Current smokers	195(43.7)	59(28.1)	
Ex-Smokers	57(12.8)	22(10.5)	
Alcohol drinking			0.853
None	344(77.5)	157(75.8)	
Current drinkers	97(21.8)	48(23.2)	
Ex-drinkers	3(0.7)	2(1.0)	
Creatinine (mean ± SD), mg/dl	2.0±2.6	1.4±2.0	0.005
Uric acid (mean ± SD), mg/dl	8.0±2.5	7.3±2.1	<0.001
HDL-C (mean ± SD), mg/dl	34.7±12.2	37.6±15.0	0.009
LDL-C (mean ± SD), mg/dl	175.4±54.0	157.9±41.7	<0.001
Cholesterol (mean ± SD), mg/dl	244.2±61.2	219.2±45.2	<0.001
Non-HDL-C (mean ± SD), mg/dl	211.8±59.3	185.9±43.1	<0.001
TRL-C (mean ± SD), mg/dl	35.4±38.3	27.5±28.8	0.006
Triglyceride [median (IQR)], mg/dl	153 (100-239)	112.0 (76-184)	<0.001

TRL-C, triglyceride-rich lipoprotein cholesterol; IQR, interquartile range.

a: numbers not equal to total number were due to missing data.

b: Student's *t*-test, Wilcoxon rank-sum test or Chi-squared test.

c: including Hakka and aborigines.

d: including retiree and housekeeper.

**Table 2:** Association between *MnSOD, CAT*, and *GPx1* genotypes and risk of coronary artery disease.

Genotype	Cases, n (%) N=447	Controls, n (%) N=210	OR(95% CI)^a^	P-value
MnSOD Val-9Ala				
Val/Val	352(78.7)	181(86.2)	1.00	
Val/Ala	83(18.6)	27(12.9)	1.82(1.10-2.99)	0.019
Ala/Ala	12(2.7)	2(0.9)	2.40(0.51-11.3)	0.269
P for trend			0.012	
Val/Val	352(78.8)	181(86.2)	1.00	
Val/Ala+Ala/Ala	95(21.2)	29(13.8)	1.86(1.15-3.01)	0.011
CAT C-262T				
C/C	403(90.2)	197(93.8)	1.00	
C/T	42(9.4)	13(6.2)	1.43(0.73-2.82)	0.299
T/T	2(0.5)	0(0.0)	—	0.982
C/C	403(90.2)	197(93.8)	1.00	
C/T+T/T	44(9.8)	13(6.2)	1.51(0.77-2.95)	0.233
GPx1 Pro198Leu				
Pro/Pro	406(90.8)	191(90.9)	1.00	
Pro/Leu	41(9.2)	19(9.1)	1.08(0.58-1.99)	0.817

a: adjusted for age, sex, cigarette smoking, and alcohol drinking.

**Table 3:** Association between *MnSOD, CAT*, and *GPx1* genotypes and risk of coronary artery disease severity.

Genotype	CAD severity			
	0	1	2	3
MnSOD				
Val/Val	181	119	102	131
Val/Ala+Ala/Ala	29	40	27	28
OR(95% CI)^a^	1.00	2.31(1.32-4.03)	1.92(1.02-3.61)	1.40(0.75-2.61)
P-value		0.003	0.043	0.288
CAT				
C/C	197	145	117	141
C/T+T/T	13	14	12	18
OR(95% CI)^a^	1.00	1.25(0.55-2.83)	1.40(0.58-3.34)	1.80(0.82-3.99)
P-value		0.592	0.454	0.146
GPx1				
Pro/Pro	191	142	120	144
Pro/Leu	19	17	9	15
OR(95% CI)^a^	1.00	1.24(0.60-2.59)	0.83(0.34-2.03)	1.05(0.49-2.28)
P-value		0.562	0.689	0.895

a: adjusted for age, sex, cigarette smoking, and alcohol drinking.

**Table 4:** Association between *MnSOD, CAT*, and *GPx1* genotypes and risk of coronary artery disease stratified by cigarette smoking status.

Genotype	Cigarette smoking^a^	Cases, n (%)	Controls, n (%)	OR(95%CI)^b^	P-value
MnSOD					
Val/Val	Never	150(33.6)	109(51.9)	1.00	
Val/Ala+Ala/Ala	Never	44(9.9)	20(9.5)	1.79(0.95-3.37)	0.073
Val/Val	Ever	201(45.1)	72(34.3)	1.00	
Val/Ala+Ala/Ala	Ever	51(11.4)	9(4.3)	2.23(1.02-4.88)	0.045
P for interaction				0.552	
CAT					
C/C	Never	174(39.0)	122(58.1)	1.00	
C/T+T/T	Never	20(4.5)	7(3.3)	1.80(0.71-4.60)	0.217
C/C	Ever	228(51.1)	75(35.7)	1.00	
C/T+T/T	Ever	24(5.4)	6(2.9)	1.20(0.46-3.12)	0.708
P for interaction				0.387	
GPx1					
Pro/Pro	Never	177(39.7)	116(55.2)	1.00	
Pro/Leu	Never	17(3.8)	13(6.2)	1.06(0.47-2.41)	0.894
Pro/Pro	Ever	228(51.1)	75(35.7)	1.00	
Pro/Leu	Ever	24(5.4)	6(2.9)	1.23(0.47-3.21)	0.674
P for interaction				0.773	

a: “Never” is nonsmokers and “Ever” is the combination of current smokers and ex-smokers.

b: adjusted for age, sex, and alcohol drinking.

**Table 5:** Association between *MnSOD* genotypes and risk of coronary artery disease severity stratified by cigarette smoking status.

	CAD severity			
	0	1	2	3
Never cigarette smoking				
MnSOD				
Val/Val	109	51	48	51
Val/Ala+Ala/Ala	20	20	14	10
OR(95% CI)^a^	1.00	2.07(0.98-4.37)	1.91(0.81-4.52)	1.45(0.58-3.62)
P-value		0.056	0.140	0.431
				
Ever cigarette smoking				
MnSOD				
Val/Val	72	67	54	80
Val/Ala+Ala/Ala	9	20	13	18
OR(95% CI)^a^	1.00	2.63(1.07-6.44)	2.11(0.81-5.54)	1.73(0.69-4.36)
P-value		0.034	0.129	0.245

a: adjusted for age, sex, and alcohol drinking.

## Data Availability

The data used to support the findings of this study were supplied by Dr. Chih-Ching Yeh under license and so cannot be made freely available. Requests for access to these data should be made to Dr. Chih-Ching Yeh by email: ccyeh@tmu.edu.tw.
